# Perceived quality of life in partners of patients undergoing treatment in somatic health, mental health, or substance use disorder units: a cross-sectional study

**DOI:** 10.1186/s12955-017-0750-5

**Published:** 2017-08-30

**Authors:** Bente Birkeland, Bente M. Weimand, Torleif Ruud, Magnhild M. Høie, John-Kåre Vederhus

**Affiliations:** 1 Sørlandet Hospital HF, Addiction Department, Research Unit, Addiction Unit, Sørlandet Hospital HF, P.b. 416, 4604 Kristiansand, Norway; 2 Akershus University Hospital, Division Mental Health Services, Lørenskog, Norway; 3 University of Oslo, Institute of Clinical Medicine, Oslo, Norway; 4 Department of Psychosocial Health, University of Agder, Faculty of Health and Sports Sciences, Grimstad, Norway

**Keywords:** Quality of life, Partner, Substance use disorder, Illness, Family cohesion, Social support, Psychological distress

## Abstract

**Background:**

This study explores (1) differences in socio-demographic, social/familial, and health variables and perceived quality of life (QoL) among partners of patients with somatic illness, mental illness, or substance use disorder (SUD); and (2) identifies factors associated with QoL.

**Methods:**

Participants (*N* = 213) in this cross-sectional study were recruited from inpatient or outpatient services in five hospitals in Norway, 2013–2014. QoL was measured by the QoL-5, a generic five-item questionnaire. Differences between groups were examined using Chi-square for categorical variables and Kruskal-Wallis for contiuous variables. Multiple linear regression analyses were used to examine factors associated with QoL.

**Results:**

The mean QoL score was similar to that of a general population sample, and 13% of the sample had a markedly low QoL. Partners in the SUD group experienced worse socio-demographic conditions in terms of occupation and income, but QoL did not differ significantly among the three groups. In a regression model, perceived family cohesion was positively associated with QoL while psychological distress (Symptom Checklist-10) was negatively related to it. The model explained 56% of the variance in QoL.

**Conclusions:**

When patients are ill, clinicians should consider the partners’ QoL, and brief QoL tools can be used to identify those who are struggling most. Reduced QoL is associated with higher psychological distress and lower family cohesion. Treatment initiatives focusing on these themes may serve as preventive measures to help the most vulnerable families cope with their difficult life situation.

## Background

An estimated 30–50% of the general population in Norway will experience a mental disorder in their lifetimes, 10–20% will experience a substance use disorder (SUD), and about 30% will experience cancer [1]. Thus, during their lifetimes, many people will experience an illness of a partner or other loved one across different illness domains. The illness not only will affect the patient but also will impact the partner, and several studies have found that the partner’s quality of life (QoL) is negatively affected and typically lower than that of the general population [2, 3]. According to some studies, partner QoL can be even lower than that of the patient [2, 4].

QoL can be affected for several reasons. A loved one’s illness may raise concerns and worries about the future [2], which in turn can lead to stress, fatigue, and sleep deprivation [2, 5]. Such factors can influence physical and mental health negatively, and anxiety and depression can be among the consequences [6–8]. Physical and mental health form two integral components of QoL [9, 10], and when they are affected, QoL will typically be perceived as impaired.

Another typical component of QoL is the social domain, or how people rate their social relations [11]. A stressful event such as the illness of a loved one can be hard to deal with especially for partners who have no one with whom to share their problems [12–14]. Conversely, higher perceived social support has consistently been associated with higher QoL [2, 3, 14, 15]. Social life within the family is often affected as well; family cohesion may become disrupted, and the partner’s familial capacity can be weakened, as when the partner’s care is directed towards the needs of the patient at the expense of the needs of children in the household [16, 17]. Several studies have reported correlations between QoL and impaired family life [2, 5, 14].

Studies also have examined the association between socio-demographic variables and the QoL of those who have an ill partner [5–7, 14, 18]. Although there is no obvious reason why gender, for example, would be associated with higher or lower QoL, some studies suggest that the QoL of female partners is more affected than that of male partners [5, 8, 14]. Likewise, other socio-demographic variables and QoL might not be expected to interact automatically – i.e., people can be content with their lives despite difficult life circumstances [9] – but adverse life conditions such as unemployment, higher financial burdens, or poverty would add to the strain when people experience stressful life events such as a loved one’s illness. Factors like these could reduce the ability to cope with the situation and in turn negatively affect QoL, as some studies also have suggested [2, 5, 12]. Lack of engagement in work and/or school activity in themselves negatively affect QoL in a normative population [19]. More specifically, partners of mentally ill patients or SUD patients often experience stigma attached to the illness or substance abuse [20, 21], and some may even report overwhelming feelings of guilt and shame [22, 23]. Such additional emotional burdens may cause partners, especially in these two illness domains [24], to withdraw from social networks, further eroding QoL.

In this study, we compared QoL in partners of patients with illness across several illness domains, including somatic health, mental health, and SUD. To our knowledge, no studies have addressed this question by comparing these domains in this way. We hypothesized that:

1. Partners of ill patients would have a lower QoL compared to the normative population.
2. Partners of patients with mental illness or SUD would have a lower QoL compared to partners of patients with somatic illnesses.

The study aims were to (1) explore differences in socio-demographic, social/familial, and health variables and perceived QoL between partner groups and (2) to identify factors associated with QoL.

## Methods

### Study setting

This study used data from a Norwegian cross-sectional, multicenter study in which the overall objective was to explore the experience of children when one of their parents has an illness, their perceived need for support, and to what extent they receive support. Both parents (the patient and the other parent) were included to give collateral information about the child and to report on their own situation during illness [25]. Thus, the main study consisted of the child, the patient with the illness, and the other biological parent who was or had been the partner of the patient. The study had a broad perspective and set out to examine the children’s situations across illness domains within the specialist health care services, i.e., in somatic health (severe neurological conditions or cancer), mental health, and SUD treatment services. Participants were recruited in five Norwegian hospitals. The present study used a subset of these data to examine the sample of partners.

#### Sample

The partners were recruited via patients in the main study (see above). The patients gave consent to inclusion of partners, and those partners willing to participate gave written informed consent. Respondents were life partners of the patients of the main study, and they shared parenthood responsibilities. As the present study focused on the QoL of partners, we did not include parents that were separated or divorced. Partners who could not read and write Norwegian were excluded.

Of the 534 families included in the main study, 266 partners or ex-partners consented to participate (50%) and 213 (40%) still remained life partner of the patient. In cases of non-inclusion, the patient did not consent to invite the partner to the study or the partner was not willing to participate. The proportion in each category is not known because of insufficient administrative routines, but the inclusion rate in the mental health and SUD illness domains was lower than in the somatic domain (36%, 19%, and 57%, respectively; χ^2^ = 51.7, *p* < 0.001).

#### Data collection

The participants were recruited from March 2013 to December 2014. Recruitment and data collection were carried out by a local coordinator and co-workers at each hospital. Data collection was conducted according to participant choice, usually at their home. Responses to questionnaires that required about one hour to complete were entered on tablets.

### Measures and procedures

We included a number of demographic and social indicator variables reflecting living conditions, which may advantageous when examining QoL [26]. In this study, age, gender, work/school status, educational level, and income were examined. Occupation and ongoing education, i.e., work/school status, was summarized into an indicator for activity (percentage engaged in work/school), following the example of Barstad [27].

#### Social and familial variables

##### Social support

As mentioned, the perceived available social support can be important for how a person copes with a stressful life event and may therefore be an important predictor for perceived QoL in such circumstances. In the analysis, we have placed “social support” and “family cohesion” (see below) under the headings “social/familial variables.” Social support was measured using the Interpersonal Support Evaluation List (ISEL), which includes 12 items on social support in both daily life and crises [28]. This instrument is an instrument used to measure the perceived availability of four subscales (Belonging Support, Self-esteem Support, Tangible Support, and Appraisal Support), and the response options range from 1 to 4, with a sum score of 12 to 48; higher scores indicate higher perceived support. There is no generally accepted cut-off for high versus low perceived social support in ISEL. The mid-point of the scale is 30, and scores above that value indicate a more positive than negative view of the amount of social support available [29]. In a US-based general population sample, the mean score was 42.7 (5.0) [30]. The instrument has been used in several countries [31, 32], including Norway [33].

##### Family cohesion

Family cohesion was measured using the Family Cohesion Subscale (FACES-III) [34, 35], with 10 items describing relations among family members and the degree to which family members feel separated from or connected to their family. The responses range from 1 to 5, with a sum score from 10 to 50 and higher scores indicating higher cohesion. A mean score of 39.8 has been found in a general population [36], and a score below 40 indicates perceived lack of cohesion. The Norwegian version has been validated and found to be usable [33].

##### Partner’s perceived family capacity

To measure whether the partner’s care was directed towards the illness of the patient at the expense of the children and partner’s own needs, the main study’s project group constructed a set of eight questions for the present investigation. The questions were informed by a qualitative study among Norwegian families with substance use problems [37]. The questions began with the phrase, “Does the condition of the ill parent affect your capacity to…” and were completed with phrases covering eight domains, e.g., “do practical housework,” “…give the children emotional support,” “…give the children structure,” and “…participate in social activities with the children.” The questions were scored on a 4-point scale (0 = not at all, 1 = slightly, 2 = to some degree, 3 = to a larger degree). A high score indicates a high influence of the condition/disease on the capacity of the partner in the family. The factor structure of this new scale was examined with an exploratory factor analysis using principal axis and an oblique rotation method (promax). Kaiser’s eigenvalue-greater-than-one rule was used to determine the number of factors, and items were retained if they had factor loadings >0.4 [38]. The analysis yielded a univariate solution, and only one factor was extracted; all items had factor loadings above 0.57, and the scale explained 67% of the variance. The Cronbach’s alpha was 0.93.

##### Concerns about child/children

The partner’s level of concern about their children, i.e., worries about their well-being and functioning, was measured using a single question: “Do you have any concerns about your child’s well-being and functioning?” The question was scaled similarly to the family capacity scale, and a higher score indicated a greater concern for the child. No normative data for this single item exist, as it has been used only in this study.

#### Health variables

##### Psychological distress

Perceived psychological distress was measured using the Hopkins Symptom Checklist 10 (SCL-10), a short-form of the Hopkins Symptom Checklist 90 [39], which has 10 items about anxiety (4 items) and depression (6 items). Responses were scored on an ordinal scale from 1 to 4, with the highest score indicating highest distress. A total mean score above 1.85 is considered a pathological score, with a mean score of 1.36 in a general population [40, 41]. The SCL-10 assessment is considered to provide a good indicator of psychological distress and is validated and considered suitable for use in Norway [40].

##### Substance use

Substance use and substance use problems in participants were measured with the CAGE questionnaire Adapted to Include Drugs (CAGE-AID) [42]. This instrument includes use of legal and illegal substances, as well as legal substances used in a way other than prescribed. A sum score based on four questions (“yes/no”) was calculated (range 0–4), and a score of 2 or higher indicates a substance use problem [42]. A mean score of 0.9 was found in a hospital population sample of non-SUD patients [43]. National guidelines for assessment of substance use in Norway recommend CAGE-AID as an assessment tool [44].

#### Quality of life (QoL)

QoL was measured with the QoL-5 [45], a generic, validated instrument covering overall QoL, based on an integrative theory of QoL and considered relevant as a disease-nonspecific instrument [45–47]. The reason for choosing the QoL questionnaire was that the instrument has been described as useful for measuring the overall QoL for both general population samples and across different illness domains [45, 46, 48]. It consists of five subjective QoL statements: two questions on mental and physical health, two questions on the quality of the relationship with important others (partner and friends), and one question regarding existential QoL, meaning relation to self. Responses are scored on a five-step ordinal scale ranging from very poor to very good QoL, and then transferred to a decimal scale from 0.1 to 0.9, where 0.9 is the highest/best score and 0.1 the lowest/worst [45, 49]. Mean scores for health, relationships, and existential QoL were calculated, as was a total QoL score as a mean of the three scores. A mean score of 0.69 was found in a previous survey of the general population and was used as our population reference [45]. The cut-off score for a markedly low QoL has been suggested to be −0.15 below the general population (<0.55) [47]. The instrument has been used in a number of studies and is sensitive to QoL changes and for capturing differences in QoL; it has also been established as a valid and reliable instrument [45, 47, 50].

### Statistical analyses

Descriptive statistics were used to present sample characteristics. Differences between groups were examined using Chi-square for categorical variables. The Kolmogorow-Smirnov test was implemented to ascertain whether continuous variables were or were not normally distributed. Since the criteria for a normal distribution were not fulfilled, the nonparametric Kruskal-Wallis test was used for the inter-group comparisons. If significant, results were followed up with a Mann-Whitney U-test. Before the multiple regression analysis, preliminary bivariate analyses were used to examine factors associated with QoL; factors with a *p* value below 0.20 were included in the following sequential procedure following the lax criterion recommended by Altman [51]. A stepwise procedure (hierarchical regression) was used to examine the relative influence on QoL of socio-demographic, social/familial, and health variables. In the first step, we included socio-demographic variables (group, gender, education, work/school activity, and income). In the second step, we included social/familial variables (family cohesion, social support, concern for child, and whether the familial capacity was influenced by the illness). Finally, in the last step, we added health variables (psychological distress). The dependent variable (QoL) was expected to be skewed toward the higher end of the scale; thus, a bootstrapping procedure (1000 replications) was used to obtain more robust estimates. Results are presented as unstandardized beta coefficients with 95% confidence intervals (CIs). The R square (R^2^) value was used to assess the fit of the statistical model. Analyses of variables were considered to be statistically significant at *p* < 0.05. All analyses were performed using IBM SPSS Statistics version 21.

## Results

### Differences between partner groups

The total sample consisted of 213 partners: 116 in the somatic illness domain, 72 in the mental illness domain, and 25 in the SUD domain (Table 1). We found significant differences across groups. The proportion of women was higher in partners in the SUD group. Partners in this group also reported having significantly lower income, lower education level, and less work/school activity.

**Table 1:** Characteristics of participants (*N* = 213), with data presented as N (%) or *mean (SD) / median (Interquartile range, IQR) [italics*]

Variables	Somatic group (A) *N* = 116	Mental illness group (B) *N* = 72	Substance use (SUD) group (C) *N* = 25	Total *N* = 213	*p* value^a^	A / B^b^	*A / C* ^b^	*B / C* ^b^
Age, years	*43 (7)/43 (9)*	*39 (8)/38 (15)*	*36 (10)/35 (15)*	*41 (8)/41 (12)*	<0.001	0.004	0.001	ns.
Gender, women	35 (30)	13 (18)	16 (64)	100 (38)	<0.001	ns.	0.001	<0.001
Work/school activity^c^	89 (26)/109 (94)	85 (33)/63 (88)	52 (46)/15 (60)	83 (33)/187 (88)	<0.001	ns.	<0.001	0.003
Educational level								
- Primary education	13 (11)	5 (7)	6 (24)	24 (11)				
- High school	37 (32)	36 (50)	14 (56)	87 (41)	0.003	0.044	0.003	0.023
- College/university	66 (57)	31 (43)	5 (20)	102 (48)				
Income^d^	*986′ (439)/900′ (380)*	*770′ (271)/700′ (250)*	*481′ (224)/450′ (210)*	*849′ (403)/800′ (385)*	<0.001	<0.001	<0.001	<0.001
Social support (ISEL)	*38.2 (6.2)/38.5 (10.8)*	*37.3 (7.3)/37.0 (10.8)*	*37.2 (6.9)/38.0 (13.0)*	*37.8 (6.7)/38.0 (10.0)*	0.767			
Family cohesion (FACES-III)	*42.3 (5.4)/43.0 (8.0)*	*41.4 (5.9)/42.0 (9.8)*	*40.6 (8.0)/41.0 (9.5)*	*41.8 (6.0)/43.0 (9.0)*	0.649			
Perceived family capacity influenced by patient’s illness^e^	*0.9 (0.8)/0.8 (1.5)*	*1.2 (1.0)/1.0 (1.8)*	*1.0 (0.9)/0.6 (1.5)*	*1.0 (0.9)/0.9 (1.5)*	0.053			
Perceived concern for children^f^	*0.9 (0.9)/1.0 (2.0)*	*1.0 (1.1)/0.0 (2.0)*	*0.4 (0.8)/0.0 (0.0)*	*0.8 (1.0)/0.0 (2.0)*	0.036	ns.	0.013	0.017
Substance use (CAGE-AID), cut-off >2	2 (2)	2 (3)	3 (12)	7 (3)	0.031	ns.	0.012	ns.
Perceived psychological distress (SCL-10)	*1.42 (0.43)/1.30 (0.68)*	*1.49 (0.58)/1.30 (0.80)*	*1.40 (0.55)/1.20 (0.40)*	*1.44 (0.50)/1.30 (0.70)*	0.877			
Quality of Life (QoL-5)	*0.72 (0.13)/0.73 (0.17)*	*0.68 (0.16)/0.68 (0.23)*	*0.73 (0.13)/0.73 (0.20)*	*0.71 (0.14)/0.70 (0.23)*	0.122			

^a^ *p* value obtained from Chi-square tests or Kruskal-Wallis

^b^When the three group tests were significant, results were followed up with paired comparisons. *p* value obtained from Chi-square or Mann-Whitney U-test. The term ns. means non-significance

^c^Percentage engaged in work/school

^d^Income in 1000 NOK

^e^Scale 0–3; higher score indicates that the condition of the ill parent had a higher impact on the other parent’s family capacity

^f^Scale 0–3; higher score indicates a higher concern for the child’s/children’s situation

The mean score on the family cohesion scale (FACES-III) was above the cut-offs for lack of cohesion and on the positive side of the social support scale (ISEL). The partners’ perceived capacity in the family was affected only modestly by the illness of the patient, as evidenced by a mean score close to the term “slightly affected” on the scale. There were no significant differences across groups in these variables. In terms of concern for the child/children in the family; the participants had little worry for the child (a mean score of ≤1 on the scale), with the lowest score in the SUD group.

In terms of health variables, only 7 (3%) scored above the cut-off the for severe substance use problems (CAGE-AID), with a slightly higher proportion of problematic substance use in the SUD partner group. Regarding perceived psychological distress (SCL-10), the mean score (1.44, SD 0.50) was below the pathological cut-off for all three groups, with 39 (18%) participants scoring above the cut-off for psychological distress. No differences in perceived psychological distress (SCL-10) emerged among the three groups.

QoL scores were similar to those of the normative population for the sample as a whole (0.71, SD 0.14), with no significant differences among groups (Table 1). A small proportion of the sample (13%) reported a markedly low QoL (<0.55).

### Variables associated with QoL

In bivariate analyses, age and substance use (CAGE-AID) had *p*-values above the recommended lax criterion (*p* > 0.2); thus, they were excluded from further analyses and from the following model.

The first step of the hierarchical regression (socio-demographic variables) (Table 2) showed that income and work/school activity were significantly associated with QoL. This model explained 6% (R^2^) of the variance. In the second step of the hierarchical regression, we added social/familial variables; family cohesion (FACES-III), perceived social support (ISEL), and perceived worry/concern about the child/children were significantly associated with QoL (Table 2). This model explained 33% (R^2^) of the variance in QoL. The final model included health variables, and only two variables were significantly associated with QoL: perceived family cohesion and psychological distress. Perceived family cohesion was positively associated with QoL while psychological distress (SCL-10) was a negative predictor (beta = −0.16; 95% CI = −0.20/−0.13, *p* < 0.001; Table 2). The final model explained 56% (R^2^) of the variance in QoL (Table 2).

**Table 2:** Factors associated with QoL (*N* = 266)

Variables	Block 1^b^		Block 2^b^		Block 3^b^	
Socio-demographic variables	B (95% CI)	*p* value^a^	B (95% CI)	*p* value^a^	B (95% CI)	*p* value^a^
Group^c^	0.02 (−0.01/0.05)	0.141	0.02 (−0.01/0.04)	0.242	0.00 (−0.02/0.03)	0.715
Gender	−0.02 (−0.07/−0.02)	0.279	−0.03 (−0.07/0.01)	0.141	−0.01 (−0.04/0.03)	0.758
Education	0.01 (−0.02/0.04)	0.431	0.01 (−0.02/0.04)	0.498	0.00 (−0.02/0.03)	0.760
Work/school activity^d^	0.00 (0.00/0.00)	0.050	0.00 (0.00/0.00)	0.241	0.00 (−0.00/0.00)	0.669
Income^e^	0.01 (0.00/0.01)	0.030	0.00 (−0.00/0.01)	0.180	0.00 (−0.00/0.01)	0.437
Social / familial variables						
Family cohesion (FACES-III)^f^			0.05 (0.02/0.08)	0.003	0.05 (0.02/0.07)	0.001
Social support (ISEL)^g^			0.07 (0.03/0.10)	0.001	0.03 (−0.00/0.06)	0.091
Concern about child Family capacity			−0.03 (−0.05/−0.01) −0.01 (−0.04/0.01)	0.002 0.186	−0.01 (−0.03/0.01) −0.01 (−0.02/0.01)	0.206 0.558
Health variables						
Psychological distress (SCL-10)					−0.16 (−0.20/−0.13)	<0.001

^a^ *p* value obtained from multivariate linear regression, presented as beta and 95% confidence interval (CI)

^b^Explained variance (R^2^): Block 1 (socio-demographic variables) = 6%; Block 2 (social/familial variables) = 33%; Block 3 (health variables) = 56%

^c^Groups: Partners of patients in three domains – somatic or mental illness or substance use disorders

^d^Percentage engaged in work/school activity

^e^Income in NOK 100,000

^f,g^Mean scores were used to facilitate interpretation of the coefficients

## Discussion

Some socio-demographic variables differed significantly among the groups in this study; partners in the SUD group differed significantly in terms of gender (being female), lower work/school activity, lower educational level, and lower income. The QoL score for the total sample was similar to that of a normative population sample, with no significant differences in QoL among groups. In a regression model, perceived family cohesion was positively associated with QoL whereas psychological distress was negatively related to it. The model explained 56% of the variation in QoL.

The normality of the QoL scores in this population was unexpected in light of the known strain of having an ill partner [8, 12, 14, 18]. Previous studies among partners to ill patients showed that if the patient received treatment, the impact on the partner’s QoL was positive [8, 52, 53]. Our participants were recruited during a treatment period for the ill parent, which may in part explain the unexpectedly high QoL in our sample. However, in the long run, treatment does not necessarily lead to a better QoL in the partner if the patient does not have a remission [6, 53, 54]. Nonetheless, 13% reported a markedly low QoL. This finding indicated that a relatively small proportion of the sample seemed to struggle more with their life situation.

The lack of significant differences in QoL between groups was also surprising and was contrary to our hypothesis. The partners in the SUD group were worse off in terms of some socio-demographic conditions; for example, they were less likely to being occupied with work or school, and had a poorer educational level and income than the other two groups, but these findings were not reflected in a poorer QoL at the group level. In general, poorer socio-demographic conditions seem to affect QoL negatively [9]; however, the subjective experience of such conditions affects QoL more than the ‘objective’ differences [55]. Thus, in line with other research [56, 57], overall QoL is more than a measurement of objective demographic conditions; it reflects how the individual relates to these conditions. An alternative explanation may be that when the patient receives treatment, a partner in the SUD group experiences a relatively greater relief from worries and burdens and perhaps perceives a temporal relief from their worries [54]. Thus, their QoL score may have been overestimated at this specific point in time.

Family cohesion were retained as significant factors associated with QoL in the final regression model. This outcome has been seen in previous studies among partners of patients with illness and accentuates the importance of perceived proximity and cohesion in close relations to retain the QoL [2, 14, 54]. The experience of instability and insecurity that partners of ill patients report may affect perceived family cohesion and also underlie the negative influence on QoL [5, 14].

Psychological distress (SCL-10) was the strongest variable explaining variations in QoL. A one-point gain (higher psychological distress) resulted in a 0.16 lower QoL-5 score in the final adjusted model, suggesting a substantial influence when applying the clinical interpretation of the scale [46]. The fit of the model was also strengthened considerably, and the explained variance in QoL increased from 33% to 56% with inclusion of this clinical variable. Feelings of hopelessness, worry, stress, and depression have been observed in partners of somatic or mentally ill patients [12, 18], as has anxiety in relatives of SUD patients [4, 6]. Such negative emotions may underlie the psychological distress reported here, which in turn strongly predicted worse QoL. High psychological distress would likely make an individual less able to cope well with a difficult situation arising when a close relative suffers from an illness. Other studies also report strong correlations between psychological distress and poor QoL [2, 6–8, 52], affirming the findings of our final model. However, with the present design, we cannot discern whether the reported psychological distress existed before the illness or was a reaction to having an illness in the family.

### Methodological considerations

The strengths of the study include an acceptable sample size and inclusion of groups of respondents who have not been compared before; previous studies tend to focus on separate domains. However, some limitations must be kept in mind. The sample size per group may not have been large enough for detecting statistical significant differences between them. Furthermore, the participants were recruited while the ill parent was in treatment, which might limit the representativeness of the findings. The participants in most benchmark studies in this field have an average age at least 10 years greater than in our study [7, 8, 12–14, 53]. The sample is therefore mainly representative of middle-aged partners and time periods when the ill parent is enrolled in treatment. Although socio-demographic variables differed among the groups, the findings indicate that we did not recruit respondents with extreme economic or social disturbances in their lives. One possible question is if those who did not participate experienced more disturbances compared to those who did [12]. The attrition analysis showed that there was a lower inclusion rate in the mental health and SUD illness domain, indicating that our results may be positively biased in these two illness domains. Further attrition analysis was not possible because administrative data on non-inclusion were insufficiently registered. The limited sample size per group also prevented us from examining whether there were different associations between independent variables and QoL across groups, i.e. with separate regression analyses for each group. In spite of the limitations, the findings provide important information about obstacles and facilitators of QoL in partners, which may be informative for further research and interventions.

### Implications

Although the findings indicate that the sample as a whole reported a QoL score in line with the general population, some respondents still reported a markedly low QoL. We suggest that such brief QoL tools can be used to capture those who are struggling most with their life situation.

## Conclusions

Treatment services should include consideration of the partners in times of illness of patients, and short QoL assessments can be one way of identifying those with a need for particular support. For these partners, the findings of the present study suggest the most important themes that clinicians should address: family cohesion and psychological vulnerability. Treatment initiatives focusing on these themes may serve as preventive measures to help the most vulnerable families cope with their difficult life situation.

## Abbreviations

CAGE-AID: CAGE (acronym) questionnaire Adapted to Include Drugs
FACES-III: Family Cohesion Subscale
ISEL: Interpersonal Support Evaluation List
QoL: quality of life
SCL: Symptom Checklist
SUD: substance use disorder
